# Adsorption and thermodynamic studies of the corrosion Inhibition effect of *Desmodium adscendens* (Swartz) extract on carbon steel in 2 M HCl

**DOI:** 10.1186/s13065-025-01541-y

**Published:** 2025-06-09

**Authors:** Awwal Abdullahi Adamu, Ogunkemi Risikat Agbeke Iyun, James Dama Habila

**Affiliations:** Department of Chemistry, Faculty of Physical Sciences, Ahmadu Bello University, Zaria, Nigeria

**Keywords:** *Desmodium adscendens*, Corrosion, Adsorption, Thermodynamics, Carbon steel

## Abstract

This study examined the thermodynamic parameters and adsorption mechanism of *Desmodium adscendens* (Swartz) extract (DAPE) as a corrosion inhibitor for carbon steel in 2.0 M HCl. DAPE’s chemical structure was analyzed using FT-IR spectroscopy, while the inhibition efficiency and corrosion rate were determined via gravimetric analysis. Thermodynamic parameters such as activation energy, enthalpy, and entropy changes were calculated using relevant equations. Adsorption isotherms were used to assess Gibbs free energy change. DAPE, according to the FT-IR spectra contained O-H, C = O, C = C, and C-O functional groups, typical of organic corrosion inhibitors. Higher concentrations of DAPE resulted in an increase in activation energy (from 45.36 to 65.26 kJmol^− 1^) and enthalpy of activation (from 42.61 to 62.51 kJmol^− 1^), along with a decrease in entropy of activation (from − 0.163 to -0.114 kJmol^− 1^), indicating the formation of a film barrier through physisorption on the metal surface. Spontaneous adsorption of DAPE was validated by negative *∆G*_*ads*_ values (-19.52 to -20.41 kJmol^− 1^), and negative *∆H*_*ads*_ (-12.55 kJmol^− 1^) revealing an exothermic process. The Langmuir isotherm was the most suitable model, with high R^2^ values (0.9982 to 0.9995) and an equilibrium constant of absorption (K_*ads*_) of 32.57 Lg^− 1^ indicating monolayer adsorption. In addition, DAPE achieved an inhibition efficiency of 87.61%, which demonstrated its promise as a potent and cost effective corrosion inhibitor for carbon steel in acidic environments.

## Introduction

Corrosion is a natural process, which converts refined metals to a more stable form, such as its oxide or hydroxide on reacting chemically or electrochemically with its environment [1]. This unique characteristic of steel to deteriorate has garnered significant focus due to its industrial relevance, which is viewed as one of the most significant constraints on the application of steel and other materials for industrial purposes [2]. Corrosion may manifest in various forms such as rust, discoloration, cracks and other structural deterioration, which reduces the service life of metals and negatively impacts operations in industrial facilities [3].

Tackling the effects of corrosion often comes with significant economic costs, with developed countries altogether expending approximately $2.5 trillion annually, or 3.4% of GDP. Emerging economies allocate a significantly larger share of their GDP to combat corrosion, emphasizing direct costs over indirect costs due to the difficulties in assessing the latter [4]. Direct costs refer to measurable expenditures such as corrosion protection, research, and replacement of components. Indirect expenses, which are more challenging to quantify, encompass environmental damage, fatalities, equipment overdesign for metal deterioration, product contamination, revenue decline from shutdowns, product loss from spills or fires, along with disputes and other legal issues that might arise from oil and gas activities [5].

Although corrosion is seen as an unavoidable phenomenon, significant research has consistently been pursued to find efficient methods to mitigate it and minimize its effects to an economically viable degree. Techniques employed include active protection through system modeling, selection of materials and design, or passive protection by isolating the material from corrosive agents [6].

Corrosion inhibitors are substances that slow down corrosion of metallic materials when added in small amounts and dispersed in a corrosive environment by preventing the electrochemical corrosion process of metallic surfaces [7]. This approach has proved effective in preventing corrosion through its interaction with active sites on the metal surface by forming a thin oxide film, or a passive adsorbed layer, or by eliminating aggressive environmental constituents via formation of complexes [8]. Extensive research is still ongoing for the most suitable and ideal corrosion inhibitor based on its availability, renewability, biodegradability, environmental friendliness, and cost effectiveness in protecting metals from corrosive environments, particularly hydrochloric and sulphuric acid, which are important mineral acids used in industrial operations [9]. Synthetic organic inhibitors have been successful in corrosion control, however focus has shifted from them due to their toxicity, posing risks to human health, the environment, and aquatic life [10], in addition to being expensive and labor-intensive prompting the research for alternative materials [11].

Studies on the viability of natural organic materials such as plant extracts have gained traction as they consist of secondary metabolites with polar or nonpolar properties such as phenolic compounds, flavonoids, vitamins, amino acids, volatile oils, tannins, alkaloids, and anthraquinones that contain heteroatoms (oxygen, nitrogen, sulfur), aromatic rings, aliphatic chains, heterocyclic rings and functional groups in their structures [12, 13]. Adsorption of inhibitor molecules typically occurs on the metal’s active surface sites, where greater electron density on the functional groups makes it simpler to form bonds during adsorption, increasing the degree of inhibition that results [14].

*Desmodium adscendens* (SW) DC is a perennial herbaceous plant member of the Fabaceae family that grow wild in tropical parts of the world, like West Africa and the Amazon region of South America [15]. It is abundant in phytochemicals, particularly flavonoids, polyphenols, and reducing sugar and also contains alkaloids, glycosides, saponins, and tannins, albeit in lower amounts compared to the former [16]. The plant has been extensively studied for its medicinal properties and effectively treats conditions such as psychosis, rheumatism, jaundice, asthma, and CNS disorders, exhibiting qualities like antioxidant, antibacterial, anti-inflammatory, and cardio-protective effects [17]. The focus on studying *Desmodium adscendens* (SW) DC arises from the pursuit of eco-friendly inhibitors that are affordable, biodegradable, non-toxic, and sustainable. Additionally, as a naturally occurring organic material that thrives as a weed with minimal economic significance in many areas of Nigeria, it offers a great chance for research, leveraging its abundance and affordability to evaluate the potential of transforming it into economically valuable products. Based on our thorough review of the literature and to the best of our understanding, no studies on corrosion involving this plant have been reported.

Since corrosion inhibition is an adsorption phenomenon, there is the need to understand the behaviour of the inhibitor on the metal of interest [18]. Adsorption isotherms unravel the impact of inhibitor concentration on surface adsorption, essential for analyzing the effectiveness of organic compounds in corrosion prevention [9]. Thermodynamics is applied in corrosion research to examine energy requirements and corrosion mechanisms with or without inhibitors [19], unravelling the interactions between metals and inhibitors.

## Materials and methods

### Materials

Carbon steel (CS), *Desmodium adscendens*, methanol (99.8%), hydrochloric acid (37%), acetone (99.8%), glass equipment, filter paper, funnel, knife, mortar and pestle, plastic container, thread, sandpapers, and masking tape. The chemicals utilized in this research were of analytical grade obtained from VWR International Ltd, and were used without additional purification.

### Carbon steel specimen preparation

The CS specimens utilized in this study exhibit the following chemical composition (in wt%): C 0.42; Mn 0.90; Si 0.32; P 0.035; S 0.04; Cr 0.90; Mo 0.20; with the balance being iron. For every experiment, square-shaped samples measuring 2.5 cm × 2 cm × 0.15 cm were utilized. The specimens were mechanically abraded using silicon carbide abrasive paper of various grits (200–600), degreased with acetone, rinsed with distilled water, dried then and ultimately stored in a desiccator until required for the experiment.

### Preparation of the plant for extraction

Plant samples of *Desmodium adscendens* were collected from Area “A” Quarters, Ahmadu Bello University Zaria, Nigeria. Following identification of the plant by Dr. Namadi Sunusi at the Department of Botany Herbarium Unit, Ahmadu Bello University Zaria, and obtaining the voucher number ABUH0285, they were cleaned, shaded for two weeks to dry, ground into powder, and stored in a plastic bag for extraction.

### Extraction and characterization of *Desmodium adscendens*

Extraction was carried out through cold maceration, using 500 g powdered *Desmodium adscendens* mixed with 1000 cm³ of 99.8% methanol. After four days of intermittent stirring, the mixture was filtered through Whatmann paper. The filtrate was concentrated via rotary evaporation at 65 °C and stored in a sealed container for corrosion studies.

Crude extract of DAPE and the adsorbed protective film layer that developed on the CS surface were individually examined through FT-IR spectroscopy employing the KBr pellet technique [20]. CS coupons were submerged in the corrosive solution containing 0.8 g/L of DAPE for 24 h, resulting in the development of a thin protective layer which was meticulously scraped from the CS surface and examined using FT-IR spectroscopy. The analysis was performed utilizing a Nicolet Is5 FTIR spectrometer (Thermo Fisher Scientific, USA) within the range of 400 cm^− 1^ to 4000 cm^− 1^.

### Corrosion inhibition investigation

A study on corrosion inhibition was performed utilizing CS coupons, which were evaluated via gravimetric methods over a range of temperatures (313–353 K) with different concentrations of DAPE (0.0 g/L– 1.0 g/L) to determine the effect of temperature on the corrosion process. In the control experiments, coupons were suspended in 250 ml beakers containing 200 cm³ of 2 M HCl. For the corrosion inhibition tests, coupons were similarly suspended but with different DAPE concentrations. Weight loss was measured over immersion duration of 360 min. Post-immersion, the coupons were cleaned, dried, and reweighed to determine weight loss (Δw) by subtracting the final weight from the initial weight. Based on the weight loss data, the corrosion rates (CR), inhibition efficiency (IE), and surface coverage (θ) were determined using the Eqs. 1, 2 and 3 respectively [21].

1 $$\:CR\:\left({gcm}^{2}{min}^{-1}\right)=\:\frac{\varDelta\:w}{At}$$

Where Δw is the weight loss, A is the total surface area of coupon, t is the immersion time.

2 $$\%\:IE=\:\frac{{CR}_{blank}\:-\:{CR}_{inhibitor}}{{CR}_{blank}}\:\times\:100$$

Where $\:{CR}_{blank}$ and $\:{CR}_{inhibitor}$ are the corrosion rates in absence and presence of inhibitor.

3 $$\:\theta\:=\:\frac{IE}{100}$$

## Results and discussion

### FT-IR characterization of DAPE extract

The spectrum of crude DAPE shown in Table 1 displays a broad peak near 3294 cm^− 1^, signifying the existence of O-H hydrogen bonding. A weak absorbance band at 3010 cm^− 1^ probably relates to C-H stretching in an aromatic ring, whereas peaks at 2853 cm^− 1^and 2923 cm^− 1^ indicate C-H stretching in sp^2^ hybridized carbon (alkenes) and sp^3^ hybridized carbon (aliphatic), respectively. A sharp peak at 1708 cm^− 1^ indicates C = O stretching. Extra peaks at 1651 cm^− 1^ and 1608 cm^− 1^ could result from the stretching of C = C bonds in alkenes and aromatic rings. A significant peak is also observed at 1455 cm^− 1^, representing C = C bending, while 1367 cm^− 1^ indicates methyl C-H bending. The peaks at 1159 cm^− 1^ and 1027 cm^− 1^ are associated with C-O stretching. In contrast, the examination of the scraped metal matrix spectrum revealed multiple matching functional groups to the crude DAPE, particularly O-H at 3334 cm^− 1^, C = C peaks appearing at 1637 cm^− 1^ and 1611 cm^− 1^ attributed to π bonds from alkenes and aromatics respectively, along with a C-O peak at 1030 cm^− 1^ that are crucial in inhibiting corrosion of CS. The intensity of the peaks were noted to appear weaker compared to crude DAPE, probably due to interaction with the metal surface, supporting the involvement of heteroatoms and π bonds that enhance its effective corrosion inhibition properties, consistent with earlier findings in the literature [12, 22, 23].

**Table 1:** FTIR for DAPE crude extract and adsorbed corrosion matrix

S/*N*	DAPE crude extract		Scraped metal matrix	
	Wavenumber (cm^− 1^)	Assigned Functional Group	Wavenumber (cm^− 1^)	Assigned Functional Group
1	3294.29	O-H	3334.80	O-H
2	3010.64	C-H Ar	2918.25	sp^3^ C-H
3	2923.41	sp^3^ C-H	2851.72	sp^2^ C-H
4	2853.47	sp^2^ C-H	1637.75	C = C alkene
5	1708.84	C = O	1611.23	C = C Ar
6	1651.33	C = C alkene	1421.28	C = C, C-H
7	1608.23	C = C Ar	1030.77	C-O
8	1513.57	C = C-C Ar	829.24	C-H
9	1455.96	C = C, C-H		
10	1367.03	C-H		
11	1159.87	C-O		
12	1027.53	C-O		
13	830.12	C-H		

### Weight loss

**Fig. 1:**
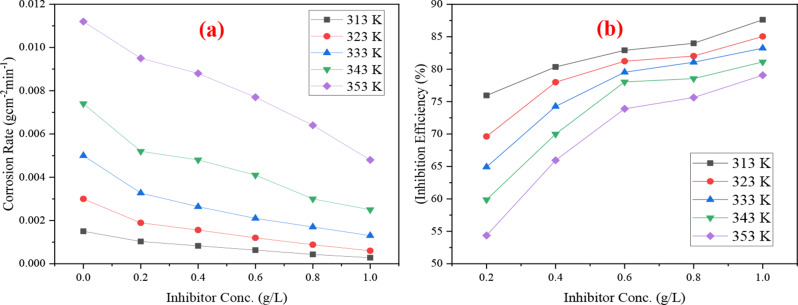
Impact of temperature on (**a**) CR and (**b**) IE at different DAPE concentrations on carbon steel in 2 M HCl

The gravimetric experiments revealed that the pace at which metals corrode is greatly influenced by temperature and inhibitor concentration [24] and thus play a crucial role in determining the corrosion rate of metals. This was confirmed for CS in 2.0 M HCl as depicted in Fig. 1a and b, which illustrates a graph of the corrosion rate and inhibition efficiency values in relation to an exposure duration of 360 min. As the DAPE concentration increases at 313 K, CR initially decreases and subsequently stabilizes, while the trend for IE is contrary to that of CR. At increased temperatures, the decrease of CR and the increase of IE persist steadily as DAPE concentration rises. However, increasing the temperature of the corrosive medium while maintaining the same DAPE concentration leads to a substantial increase in CR and a decrease in IE. These alterations might indicate the DAPE molecules ability to adsorb and create a protective film on CS surface grows as concentration levels rise, resulting in greater thickness. Nonetheless, once the adsorption sites on the metal are depleted and the system reaches saturation, the adsorption capacity will essentially stay constant, leading to CR and IE being nearly unaffected for increased DAPE concentrations.

The lowest CR of 0.00028 g.cm^− 2^ .min^− 1^ and highest IE of 87.61% were observed at lowest temperature of 313 K and highest inhibitor concentration of 1.0 g/L. This might be attributed to the fact that elevating the HCl solution’s temperature increases molecular kinetic energy, leading to more effective collisions with the CS surface, thus accelerating hydrogen evolution. This process hinders the adsorption of extract molecules onto the metal, which can also cause desorption, complicating the formation of a stable DAPE protective barrier, especially at low inhibitor concentrations [20, 25]. Consequently, this results in an increased corrosion rate of CS and decreased inhibiting effectiveness of DAPE, linking CR and IE closely to the corrosive environment’s temperature and extract inhibitor concentration.

### Thermodynamic studies

Thermodynamic characteristics including activation energy (E_a_), enthalpy of activation (∆H*), and entropy of activation (∆S*) were analyzed to assess the effect of temperature on the CS dissolution process within corrosion systems.

Activation energy (E_a_) is crucial in determining the feasibility of corrosion reactions, with lower values indicating simpler reactions and higher values imply the opposite. Corrosion inhibitors increase E_a_ by forming a barrier, thus limiting corrosion on metals [26]. Enthalpy changes (ΔH) help in assessing adsorption models for inhibitors; positive ΔH indicates endothermic reactions while negative ΔH shows exothermic reactions. A ΔH around 100 kJ/mol suggests chemical adsorption, while below 80 kJ/mol indicates physical adsorption. Entropy changes (ΔS) reflect the disorder at metal surfaces, with increased inhibitor doses leading to greater disorder during reactant transfer [20, 27].

**Fig. 2:**
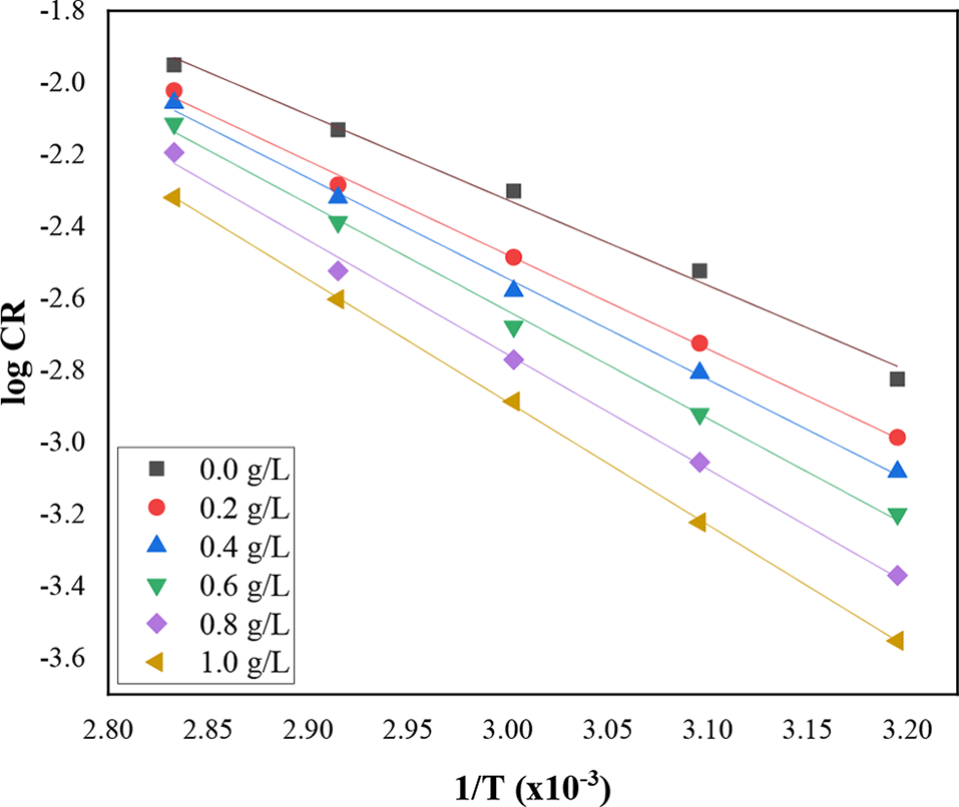
Arrhenius plot for carbon steel corrosion in 2 M HCl in absence and presence of various concentration of DAPE

The Arrhenius equation was used to assess the activation energy of corrosion for CS in acidic environments with DAPE, calculated using the following equation;

4 $$\:\text{log}CR=\text{log}A\:-\left(\frac{{E}_{a}}{2.303RT}\right)\:$$

where E_a_ represents the activation energy, R denotes the universal gas constant, and A is the factor in the exponential function. Figure 2 depicts Arrhenius plot showing the gradient of the graph of log CR against 1/T as a straight line from which the slope used to calculate the activation energy.

**Fig. 3:**
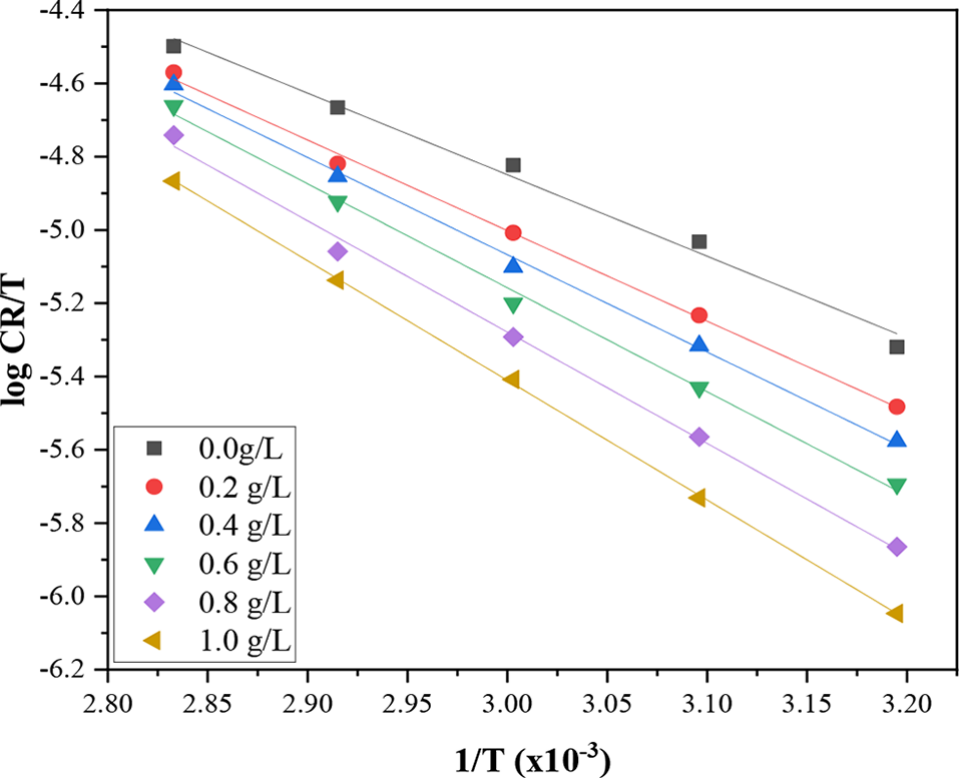
Transition-state plots for carbon steel corrosion in 2 M HCl in the absence and presence of various concentrations of DAPE

In a similar manner, the activation enthalpy (∆H*) and entropy (∆S*) were assessed using the Arrhenius equation in the transition state, as shown in the following equation;

5 $$\:\text{log}\frac{CR}{T}=\:-\frac{{\varDelta\:H}^{\text{*}}}{2.303R}\left(\frac{1}{T}\right)+\left[\text{log}\frac{R}{Nh}+\left(\frac{{\varDelta\:S}^{\text{*}}}{2.303R}\right)\right]$$

with h denoting Planck’s constant, N representing Avogadro’s number, ∆S* as the activation entropy, and ∆H* as the activation enthalpy. The plot of log CR/T against 1/T derived from the transition state equation resulted in a straight line, as illustrated in Fig. 3, allowing the assessment of the enthalpy change, ∆H*, and the entropy change, ∆S*, derived from the slope $\:-\frac{{\varDelta\:H}^{*}}{2.303R}$ and the intercept $\:\left[\text{log}\frac{R}{Nh}+\left(\frac{\varDelta\:S}{2.303R}\right)\right]$ respectively.

The activation parameters displayed in Table 2 indicated that adding DAPE increases the activation energy, creating a higher energy barrier in the corrosion reaction compared to an uninhibited solution. This increase in activation energy (E_a_) can be attributed to the physical adsorption of inhibitor molecules on the CS surface. Previous studies suggests activation energy values below 80 kJ/mol imply physical adsorption. The current study’s findings, with E_a_ ranging from 45.36 to 65.26 kJ/mol, support this assertion [21, 28, 29].

Positive ∆H* indicated that steel dissolution is an endothermic process, absorbing heat and increasing the dissolution rate at elevated temperatures [20, 30]. The study also revealed that energy of activation values exceed enthalpy values, correlating with findings by [19], suggesting corrosion involves a gas reaction, notably hydrogen evolution, reducing overall reaction volume.

**Table 2:** Fitting results for activation parameters E_a,_∆H* and ∆S*

Inhibitor Conc. (g/L)	E_a_ (kJmol^− 1^)	∆H* (kJmol^− 1^)	∆S* (kJmol^− 1^)
0.0	45.36	42.61	-0.163
0.2	50.05	47.33	-0.151
0.4	53.62	50.86	-0.142
0.6	57.10	54.35	-0.128
0.8	60.91	58.15	-0.124
1.0	65.26	62.51	-0.114

At all concentrations of the inhibitor, ∆S* was negative, signifying system orderliness, although this trend was noted to diminish at higher inhibitor levels, leading to greater entropy. This phenomenon may stem from competition between water molecules and protonated extract ions for metal surface adsorption, enhancing adsorption and desorption frequencies at increasing concentration of DAPE. Furthermore, the disorderliness created by this competition exceeds the orderliness established by the adsorption of DAPE molecules, suggesting that the rate-determining step involves dissociation, which elevates disorder from reactants to the activated complex [20, 27, 30].

### Adsorption isotherms and parameters

Adsorption isotherms, including El–Awady, Temkin, Langmuir, and Freundlich, were analyzed to understand DAPE’s adsorption on CS in HCl. Surface coverage from gravimetric experiments presented in Table 3 was fitted to these isotherms, using the coefficient of determination (R^2^) for model evaluation.

A steady temperature allows the amount of adsorbate on the adsorbent to relate to its concentration, achieving equilibrium when adsorbate molecules interact sufficiently with the adsorbent, as described by the isotherm models [25]. This offered a deeper understanding of how DAPE interacts with the metal surface at the metal-inhibitor interface under different temperatures.

**Table 3:** Gravimetric experimental data on corrosion Inhibition of carbon steel at different DAPE concentrations and temperatures in 2 M HCl solution

Inhibitor Conc. (g/L)	313 K		323 K		333 K		343 K		353 K	
	IE (%)	θ	IE (%)	θ	IE (%)	θ	IE (%)	θ	IE (%)	θ
0.0	--	--	--	--	--	--	--	--	--	--
0.2	75.94	0.76	69.63	0.78	64.92	0.75	59.87	0.72	54.36	0.7
0.4	80.34	0.8	77.99	0.79	74.26	0.78	69.99	0.75	65.95	0.73
0.6	82.90	0.83	81.23	0.81	79.53	0.80	78.04	0.78	73.89	0.74
0.8	83.99	0.84	82.02	0.82	81.07	0.81	78.54	0.79	75.63	0.76
1.0	87.61	0.88	85.04	0.85	83.23	0.83	81.11	0.81	79.07	0.79

### Langmuir isotherm

**Fig. 4:**
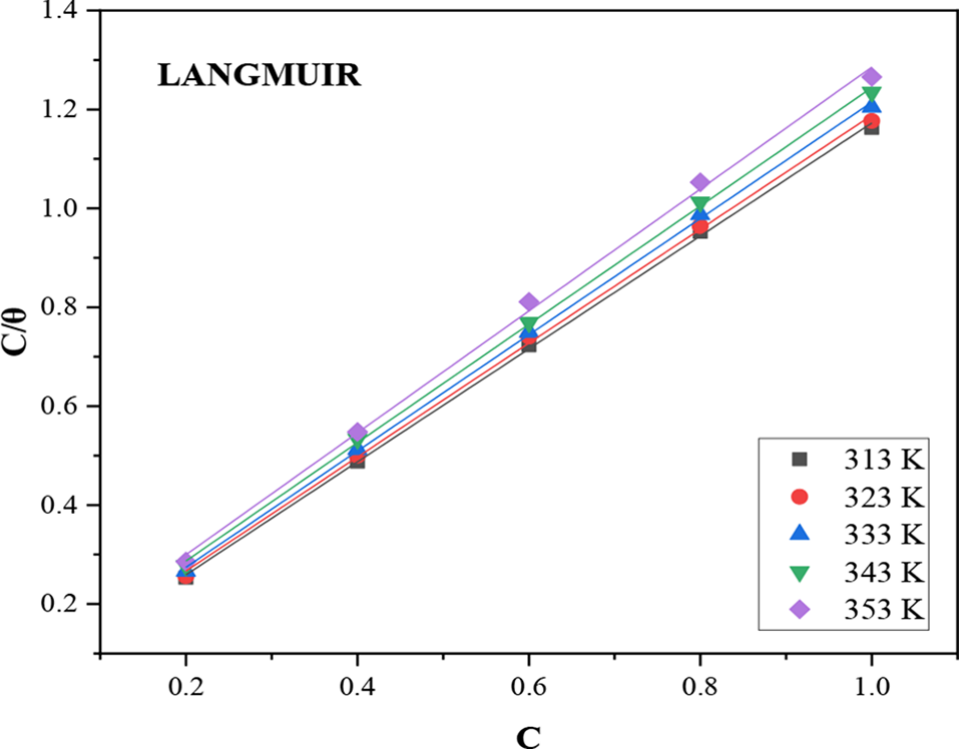
Langmuir adsorption isotherm of DAPE on carbon steel surface in 2 M HCl at different studied temperatures

Langmuir isotherm describes monolayer adsorption maximizing adsorbent capacity, based on fixed reaction sites, uniform energy, monolayer adsorption, and no interactions among adsorbed molecules [31]. The following equation can be used to express the Langmuir isotherm [32, 33]

6 $$\:\raisebox{1ex}{$C$}\!\left/\:\!\raisebox{-1ex}{$\theta\:$}\right.=\:\raisebox{1ex}{$1$}\!\left/\:\!\raisebox{-1ex}{${K}_{ads}$}\right.+C\:$$

where *C* is the concentration of the inhibitor,$$\:\:\theta\:$$ is the degree of surface coverage and *K*_*ads*_ is the adsorption constant. By plotting $$\:\raisebox{1ex}{$C$}\!\left/\:\!\raisebox{-1ex}{$\theta\:$}\right.$$ versus *C*, a straight line was obtained as shown in Fig. 4, and the value of *K*_*ads*_ evaluated from the intercept.

The adsorption parameters from Table 4 align strongly with the Langmuir adsorption isotherm model, exhibiting high correlation coefficients (between 0.9982 and 0.9995). This indicates a monolayer adsorption on the metal surface at sites of equivalent energies [31, 34]. The peak K_*ads*_ value of 32.57 Lg^− 1^ at 313 K demonstrates strong adherence of the extract to the CS surface. However, increasing temperature reduces the K_*ads*_ value as the hydrogen evolution reaction intensifies, resulting in the mechanical stripping of hydrogen atoms and decreased extract adsorption capacity [20, 25].

**Table 4:** Langmuir isotherm fitting results

Temperature (K)	*R* ^2^	Intercept	Slope	K_ads_ (Lg^− 1^)
313	0.9995	0.0307	1.1419	32.57
323	0.9991	0.0363	1.1520	27.55
333	0.9995	0.0391	1.1756	25.58
343	0.9995	0.0476	1.1965	21.01
353	0.9982	0.0531	1.2325	18.53

### Freundlich isotherm

**Fig. 5:**
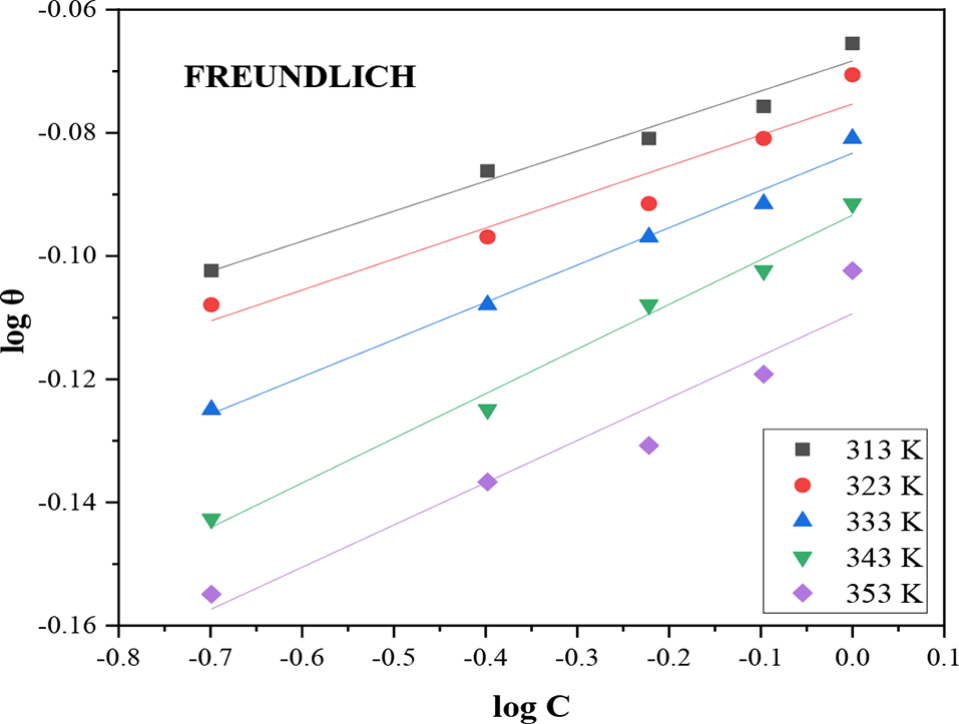
Freundlich adsorption isotherm of DAPE on carbon steel surface in 2 M HCl at different studied temperatures

The Freundlich isotherm describes non-ideal systems with weak molecular interactions throughout the physical adsorption process, forming multiple layers on heterogeneous adsorption sites with varying energies [31]. The equation below represents the Freundlich isotherm [9, 25, 35];

7 $$\:{log}\theta\:={log}{K}_{ads}+\frac{1}{n}log\:C$$

where $\:\frac{1}{n}$ is the Freundlich isotherm constant, *θ* is the degree of surface covering, *K*_*ads*_ is the adsorption constant, and C is the inhibitor concentration. A straight line with an intercept equal to log *K*_*ads*_ and a slope equal to $\:\frac{1}{n}$ is obtained by plotting the values of *log C* versus *log θ* as shown in Fig. 5.

A favorable adsorption process can be described as 0 < $\:\frac{1}{n}$ < 1 and an unfavorable adsorption process as $\:\frac{1}{n}$ > 1.

The Freundlich model values in Table 5 illustrated good fitting of corrosion data, with R^2^ in the range of 0.9308 to 0.9895. The slope for adsorption intensity (1/n) ranged from 0.0488 to 0.0686, indicating favorable and straightforward adsorption, and observed to increase with temperature highlighting increased heterogeneity of the CS adsorbent’s surface at higher temperatures [25, 35].

**Table 5:** Freundlich isotherm fitting results

Temperature (K)	*R* ^2^	Intercept	Slope (1/*n*)	K_ads_ (Lg^− 1^)
313	0.9723	0.0683	0.0488	1.17
323	0.9308	0.0753	0.0503	1.19
333	0.9895	0.0833	0.0606	1.21
343	0.9883	0.0934	0.0725	1.24
353	0.9330	0.1094	0.0686	1.29

### El-Awady isotherm

**Fig. 6:**
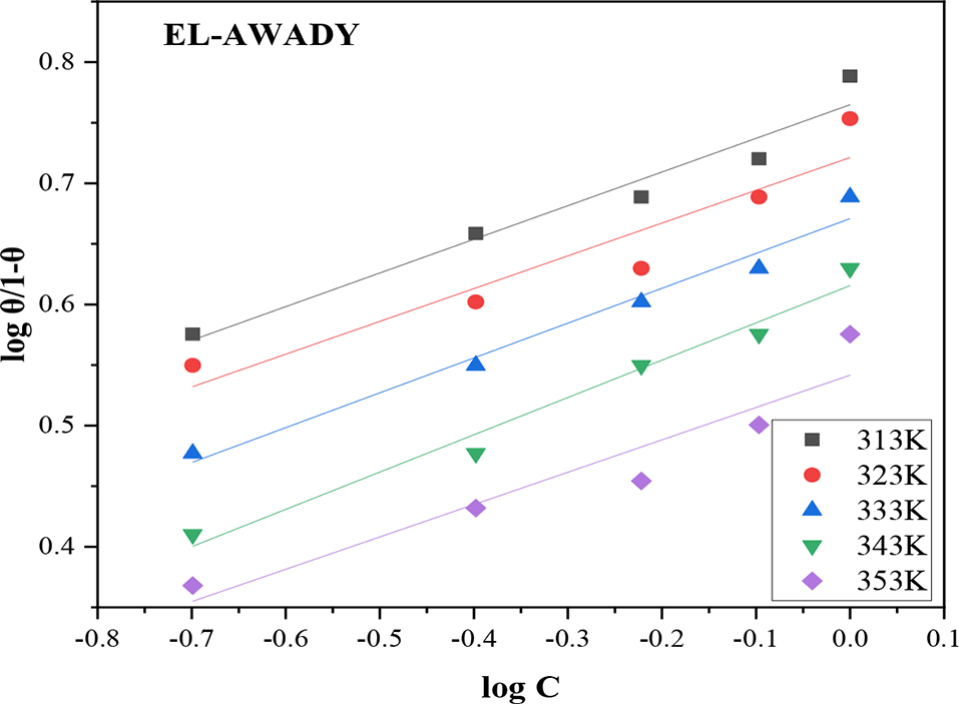
El-Awady adsorption isotherm of DAPE on carbon steel surface in 2 M HCl at different studied temperatures

The kinetic-thermodynamic El-Awady isotherm can be utilized to analyze the quantity of water molecules that can be substituted by a single inhibitor molecule on the metal surface. Stronger inhibitor-surface interaction than water leads to inhibitor molecule adsorption and vice versa [30, 35, 36]. The isotherm model equation can be written as shown below;

8 $$\:log\left(\raisebox{1ex}{$\theta\:$}\!\left/\:\!\raisebox{-1ex}{$1-\theta\:$}\right.\right)=log{K}_{ads}+ylogC$$

where *y* is the number of inhibitor particles occupying a single active site of the metal surface, *θ* is the degree of surface covering, and C is the inhibitor concentration. Plotting$$\:\:log\left(\raisebox{1ex}{$\theta\:$}\!\left/\:\!\raisebox{-1ex}{$1-\theta\:$}\right.\right)$$ vs. $$\:logC\:$$yields a straight line when the isotherm is satisfied as presented in Fig. 6. A value of 1/*y* < 1 indicates that the inhibitor forms many layers on the metal surface, whereas a value of 1/*y* > 1 indicates that the inhibitor molecule occupies multiple active sites.

The findings from the fitting in Table 6 of El-Awady isotherm show that the model had respectable correlation with the data, having R^2^ values between 0.8991 and 0.9776. In addition, the slope of 1/y values ranged from 3.25 to approximately 3.74, indicating that a DAPE molecule occupies several active sites, corroborating the results presented by [25, 35], and [20].

**Table 6:** El-Awady isotherm fitting results

Temperature (K)	*R* ^2^	Intercept	Slope (y)	1/y	K_ads_ (Lg^− 1^)
313	0.9545	0.7649	0.2778	3.5997	5.82
323	0.8991	0.7213	0.2705	3.6969	5.26
333	0.9762	0.6709	0.2876	3.4771	4.69
343	0.9776	0.6156	0.3078	3.2489	4.13
353	0.9027	0.5417	0.2671	3.7439	3.48

### Temkin isotherm

**Fig. 7:**
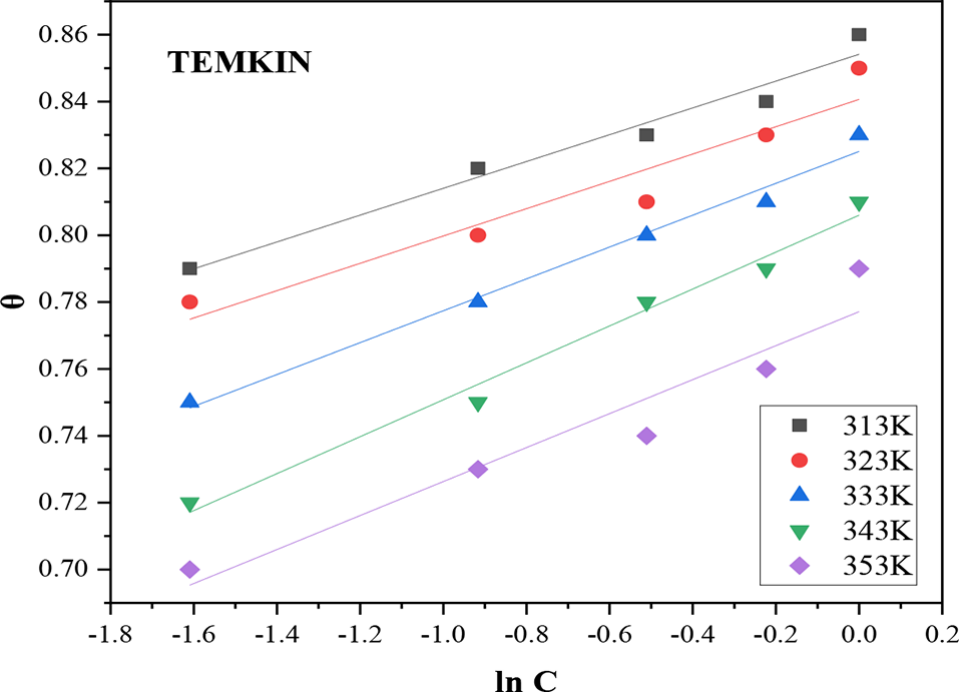
Temkin adsorption isotherm of DAPE on carbon steel surface in 2 M HCl at different studied temperatures

The Temkin isotherm considers the influence of interactions between adsorbates on the adsorption process. It is based on assumptions of uniform binding energy distribution on the adsorbent surface, linear decrease in adsorption heat with surface coverage, and interactions between adsorbate and adsorbent [31]. Temkin isotherm is shown by the following Eq. (20);

9 $$\:\theta\:=\:-\raisebox{1ex}{$1$}\!\left/\:\!\raisebox{-1ex}{$2\alpha\:$}\right.lnC\:-\:\raisebox{1ex}{$1$}\!\left/\:\!\raisebox{-1ex}{$2\alpha\:$}\right.ln{K}_{ads}$$

where *C* represents the inhibitor concentration (g/L), *θ* is a linear function of $\:lnC$, *K*_*ads*_ is the adsorption equilibrium constant, and $\:\alpha\:$ is the attractive parameter. Plotting $\:\theta\:$ versus $\:lnC$ yields a straight line when the isotherm is followed as depicted in Fig. 7. The intercept of the plot was used to calculate the equilibrium constant of adsorption (K_*ads*_) at different temperatures, while the slope was used to determine the values of the attractive parameter (α) and predict the presence of attractive or repulsive forces in the adsorption layer.

The Temkin model detailed in Table 7 demonstrated good fitting with the experimental data, showing high R^2^ value (0.9235–0.9868). The adsorption parameter α was noted to be in the negative range (-12.47 to -9.06) and decreased with rising temperature. This indicates that repulsive forces exist among the inhibitor molecules on the steel surface, but their intensity weakens as temperature increases, likely due to some inhibitor molecules being desorbed from the layer [20, 30].

**Table 7:** Temkin isotherm fitting results

Temperature (K)	*R* ^2^	Intercept	Slope	α	K_ads_ (Lg^− 1^)
313	0.9636	0.8541	0.0401	-12.47	2.349
323	0.9240	0.8406	0.0409	-12.23	2.318
333	0.9868	0.8251	0.0477	-10.48	2.282
343	0.9856	0.806	0.0552	-9.06	2.238
353	0.9235	0.7771	0.0508	-9.84	2.175

### Gibbs free energy of adsorption (*∆G*_*ads*_)

In the context of corrosion inhibition, Gibbs free energy (ΔG) is essential for comprehending the thermodynamics involved in the adsorption of inhibitors on metal surfaces. The effectiveness of an inhibitor in preventing corrosion is determined by its adsorption, while ΔG offers information on the spontaneity, intensity, and type of adsorption interactions. Basically, a negative ***∆G***_***ads***_ value indicates a spontaneous adsorption process while a positive ***∆G***_*ads*_ denotes non-spontaneity [19, 21].

The electrostatic interaction between a charged metal surface and a corrosion inhibitor results in ***∆G***_***ads***_ values reaching up to -20 kJ/mol for physisorption, whereas values lower than − 40 kJ/mol suggest chemisorption, involving the formation of coordination bonds between the metal and corrosion inhibitor species via charge sharing or transfer [20, 34].

The values of *K*_*ads*_ obtained from adsorption isotherms are related to Gibbs energy by the following equation;

10 $$\:{K}_{ads}=\:\frac{1}{C}\text{exp}\left(\raisebox{1ex}{${\varDelta\:G}_{ads}$}\!\left/\:\!\raisebox{-1ex}{$RT$}\right.\right)$$

It can be rearranged as;

11 $$\:{\varDelta\:G}_{ads}=\:-RTln55.5{K}_{ads}$$

where T is the temperature (K), R is the universal gas constant (8.314 JK^− 1^mol^− 1^), 55.5 is the concentration of water (ml/L) and ***∆G***_***ads***_ is the Gibbs free energy of adsorption calculated based on the Langmuir adsorption isotherm that had the best fit to the experimental data in Table 4 [33, 37] and presented in Table 8.

Also, the enthalpy of adsorption (***∆H***_***ads***_) and entropy of adsorption (***∆S***_***ads***_) were calculated using the basic thermodynamic Eqs. [29, 38–40];

12 $$\:{\varDelta\:G}_{ads}=\:{\varDelta\:H}_{ads}-\:{T\varDelta\:S}_{ads}$$

**Fig. 8:**
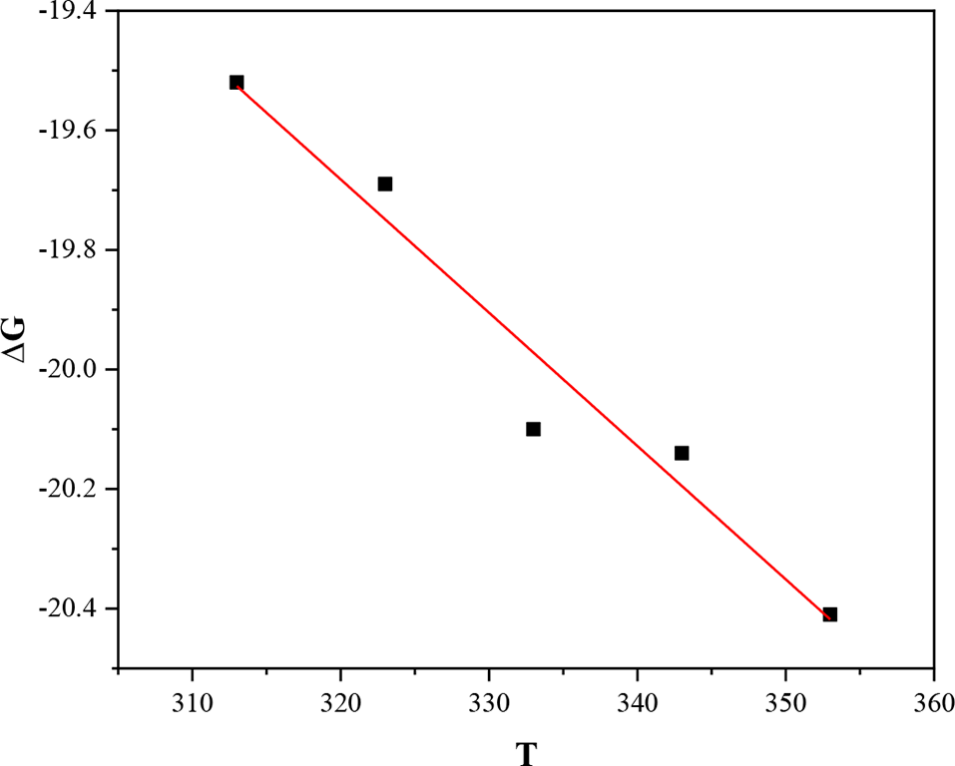
Graphical plot for the Gibbs free energy versus temperature

The intercept of the graph between the ***∆G***_***ads***_ and absolute temperature T as shown in Fig. 8 gives the values of ***∆H***_***ads***_. Values of ***∆S***_***ads***_ were obtained by putting the values of ***∆H***_***ads***_ in Eq. 12 at different temperatures.

According to the adsorption parameters in Table 8, ***∆G***_***ads***_ ranged from − 19.52 to -20.41 kJmol^− 1^ at temperatures between 313 and 353 K, indicating spontaneous adsorption mainly driven by physical mechanisms with negative ***∆G***_***ads***_ below − 20 kJmol^− 1^ as reported in studies by [27] and [41]. In addition, the values of the Gibb’s free energy obtained at 313 K were marginally lower than the values at 353 K. This could indicate that a slight rise in temperature marginally boosted the spontaneity of the adsorption process. A comparable outcome was reported by [25] when methanol extract of *Rosmarinus officinalis* (L.) was used for corrosion inhibition of aluminum alloy in 0.25 M HCl solution.

During an adsorption process, ***∆H***_***ads***_ values are helpful in anticipating physisorption or chemisorption. Physisorption typically has ***∆H***_***ads***_ values below 40 kJ/mol, whereas chemisorption is closer to 100 kJ/mol, according to various researchers [1, 29, 42]. The negative adsorption enthalpy ***∆H***_***ads***_ of -12.55 kJmol^− 1^ shows that energy in form of heat is released during the exothermic adsorption process, supporting physisorption as shown in studies by [40] and [41]. These findings align well with the decrease in inhibition efficiency as temperature is increased.

The positive values of ***∆S***_***ads***_ (0.0023 kJ/mol) highlighted the disordered nature of the system during the adsorption process, likely due to the combined effect of concurrent adsorption of organic molecules and the desorption of water molecules on the metal surface. Another potential explanation could be the exothermic characteristics of the adsorption process, which emits energy to the surrounding area, thereby increasing solvent entropy and adding to the total entropy of adsorption [37].

**Table 8:** Fitting results for adsorption parameters *∆G*_*ads*,_*∆H*_*ads*_ and *∆S*_*ads*_

Temperature	∆G_ads_ (kJmol^− 1^)	∆H_ads_ (kJmol^− 1^)	∆S_ads_ (kJmol^− 1^K^− 1^)
313	-19.52 ± 1.324E-15	-12.55 ± 0.923	0.022 ± 0.00294
323	-19.69 ± 2.468E-15		0.022 ± 0.00285
333	-20.10 ± 5.537E-15		0.023 ± 0.00277
343	-20.14 ± 5.293E-15		0.022 ± 0.00269
353	-20.41 ± 2.935E-14		0.022 ± 0.00262

## Conclusion

Thermodynamic investigations uncovered that the dissolution of CS in the presence of DAPE occurred through an endothermic process with higher activation energy in an organized corrosion system, owing to the protective film of DAPE on the metal surface that adhered via a physisorption mechanism. This could be attributed to the organic components in DAPE that were found to contain O-H, C = O, C = C, and C-O groups, indicating the existence of heteroatoms, unsaturated and aromatic compounds, which are common markers of an effective organic corrosion inhibitor. The adsorption studies validated this adsorption mechanism, which was spontaneous and exothermic in a disordered corrosion system. Langmuir isotherm model provided the best fit, indicating a monolayer adsorption while data from Freundlich, El-Awady and Temkin models described the adsorption as favorable, involving several active sites per inhibitor molecule with internal repulsive forces within the inhibitor layer, producing an inhibition efficiency of 87.61% at 1.0 g/L. Overall, the results of this study show DAPE to be an environmentally friendly material derived from nature, with significant potential as a cost-effective green inhibitor for preventing corrosion in diverse industrial applications such as protecting internal surface of oil well casing pipes and crude oil storage tanks.

## Data Availability

All data generated or analyzed during this study are included in this published article.
